# Axon hyperexcitability in the contralateral projection following unilateral optic nerve crush in mice

**DOI:** 10.1093/braincomms/fcac251

**Published:** 2022-10-03

**Authors:** Nolan R McGrady, Joseph M Holden, Marcio Ribeiro, Andrew M Boal, Michael L Risner, David J Calkins

**Affiliations:** Department of Ophthalmology and Visual Sciences, Vanderbilt Eye Institute, Vanderbilt University Medical Center, AA7103 MCN/VUIIS, 1161 21st Ave. S., Nashville, TN 37232, USA; Department of Ophthalmology and Visual Sciences, Vanderbilt Eye Institute, Vanderbilt University Medical Center, AA7103 MCN/VUIIS, 1161 21st Ave. S., Nashville, TN 37232, USA; Department of Ophthalmology and Visual Sciences, Vanderbilt Eye Institute, Vanderbilt University Medical Center, AA7103 MCN/VUIIS, 1161 21st Ave. S., Nashville, TN 37232, USA; Department of Ophthalmology and Visual Sciences, Vanderbilt Eye Institute, Vanderbilt University Medical Center, AA7103 MCN/VUIIS, 1161 21st Ave. S., Nashville, TN 37232, USA; Department of Ophthalmology and Visual Sciences, Vanderbilt Eye Institute, Vanderbilt University Medical Center, AA7103 MCN/VUIIS, 1161 21st Ave. S., Nashville, TN 37232, USA; Department of Ophthalmology and Visual Sciences, Vanderbilt Eye Institute, Vanderbilt University Medical Center, AA7103 MCN/VUIIS, 1161 21st Ave. S., Nashville, TN 37232, USA

**Keywords:** traumatic optic neuropathy, glaucoma, retinal ganglion cells, hyperexcitability, degeneration

## Abstract

Optic neuropathies are characterized by degeneration of retinal ganglion cell axonal projections to the brain, including acute conditions like optic nerve trauma and progressive conditions such as glaucoma. Despite different aetiologies, retinal ganglion cell axon degeneration in traumatic optic neuropathy and glaucoma share common pathological signatures. We compared how early pathogenesis of optic nerve trauma and glaucoma influence axon function in the mouse optic projection. We assessed pathology by measuring anterograde axonal transport from retina to superior colliculus, current-evoked optic nerve compound action potential and retinal ganglion cell density 1 week following unilateral optic nerve crush or intraocular pressure elevation. Nerve crush reduced axon transport, compound axon potential and retinal ganglion cell density, which were unaffected by intraocular pressure elevation. Surprisingly, optic nerves contralateral to crush demonstrated 5-fold enhanced excitability in compound action potential compared with naïve nerves. Enhanced excitability in contralateral sham nerves is not due to increased accumulation of voltage-gated sodium channel 1.6, or ectopic voltage-gated sodium channel 1.2 expression within nodes of Ranvier. Our results indicate hyperexcitability is driven by intrinsic responses of αON-sustained retinal ganglion cells. We found αON-sustained retinal ganglion cells in contralateral, sham and eyes demonstrated increased responses to depolarizing currents compared with those from naïve eyes, while light-driven responses remained intact. Dendritic arbours of αON-sustained retinal ganglion cells of the sham eye were like naïve, but soma area and non-phosphorylated neurofilament H increased. Current- and light-evoked responses of sham αOFF-sustained retinal ganglion cells remained stable along with somato-dendritic morphologies. In retinas directly affected by crush, light responses of αON- and αOFF-sustained retinal ganglion cells diminished compared with naïve cells along with decreased dendritic field area or branch points. Like light responses, αOFF-sustained retinal ganglion cell current-evoked responses diminished, but surprisingly, αON-sustained retinal ganglion cell responses were similar to those from naïve retinas. Optic nerve crush reduced dendritic length and area in αON-sustained retinal ganglion cells in eyes ipsilateral to injury, while crush significantly reduced dendritic branching in αOFF-sustained retinal ganglion cells. Interestingly, 1 week of intraocular pressure elevation only affected αOFF-sustained retinal ganglion cell physiology, depolarizing resting membrane potential in cells of affected eyes and blunting current-evoked responses in cells of saline-injected eyes. Collectively, our results suggest that neither saline nor sham surgery provide a true control, chronic versus acute optic neuropathies differentially affect retinal ganglion cells composing the ON and OFF pathways, and acute stress can have near-term effects on the contralateral projection.

## Teaser

Acute and progressive neurodegeneration can impact tissues unaffected by the initial insult or locus of disease. McGrady *et al*. find that axons of αON-sustained retinal ganglion cells initiate hyperexcitability in the optic nerve after crush injury in the opposing nerve. Thus, contralateral hyperexcitability could be an early indicator of neurodegeneration in the visual pathways to the brain.

## Graphical Abstract

**Graphical abstract:**
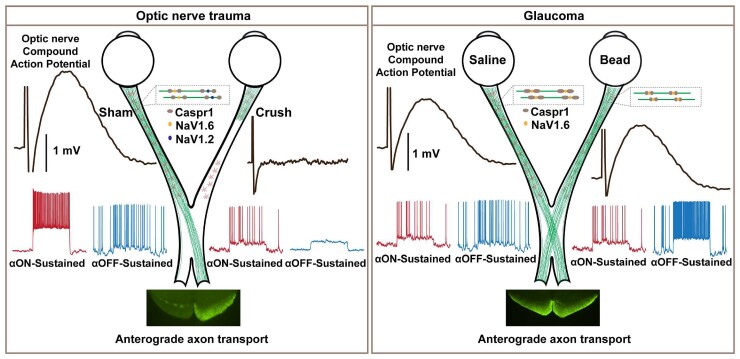

## Introduction

Tissue damage caused by neurodegenerative diseases and acute conditions is often regionally confined during early progression. Over time, however, degeneration can spread secondarily to surrounding regions. For example, clinical and experimental evidence indicate ischaemic stroke initially affects a central core. Neighbouring tissues then react, redistributing resources to restore homeostasis to the core at the expense of depleting local reserves, which effectively increases the infarct area.^1,2^ Similarly, glaucomatous optic neuropathy (glaucoma) initially attacks select retinal ganglion cell (RGC) axons that compose the superior and inferior poles of the optic nerve, causing deficits in mid-peripheral visual fields.^3^ Visual field deficits gradually expand along with increased RGC axon degeneration.^4^ Typically, glaucoma occurs bilaterally but often develops asymmetrically where the eye dominated by disease progresses more rapidly than the contralateral eye.^5^ This asymmetrical loss in visual field sectors appears to be directed by central mechanisms to maintain intact binocular fields.^6^ This clinical evidence suggest nerve damage spreads locally within and between optic projections, and retrograde signals direct compensatory mechanisms to maintain function. Similarly, in experimental glaucoma, unilateral intraocular pressure (IOP) elevation and optic nerve stress activate astrocytes and innate immune cells in the contralateral retina and projection.^7,8,9,10,11,12^ Although the contralateral retina and projection indicate a pro-inflammatory response during unilateral IOP elevation, the overall RGC activity remains intact.^10^ Even short-term unilateral IOP elevation produces a compensatory responses where astrocytes of the contralateral nerve donate metabolic resources to the stressed nerve, rendering the uninjured nerve susceptible to degeneration when subsequently stressed.^13^ Modelling traumatic insult, unilateral optic nerve crush (7–9 days) downregulates the RGC-specific transcription factor Brn3a, upregulates caspase-3, significantly reduces RGC density in the contralateral retina, though central neuron density remains stable.^7–14^ This line of evidence indicates stress is not limited to the projection directly affected and distal structures may persist during degeneration.^15^

Here, we sought to compare the pathophysiology of murine RGCs and their axons in directly injured versus contralateral tissues following optic nerve crush or induced IOP elevation by injection of microbeads.^16^ We examined RGC axon transport to the superior colliculus (SC), excitatory signalling in the optic nerve assessed through compound action potential, and RGC light- and current-evoked responses and dendritic arbour morphology. Optic nerve crush significantly reduced RGC dendritic complexity, impaired axon transport and diminished the compound action potential in the affected nerve compared with IOP elevation. Overall, we found both optic nerve crush and IOP elevation affected tissues of the contralateral projection compared with naïve. Interestingly, optic nerve crush increased excitability in the contralateral sham nerve, despite reduced expression of voltage-gated Na^+^ (NaV) channel isoform NaV1.6. We also observed contralateral hyperexcitability in large-field RGCs that produce a sustained response to light increments (i.e. αON-sustained RGCs) but not in RGCs signalling light offset (αOFF-sustained RGCs). Based on our results from light- and current-evoked responses of αON-sustained RGCs, hyperexcitability appears independent of pre-synaptic mechanisms. These results suggest unilateral optic nerve crush piques mechanisms in the contralateral nerve that retrogradely influence voltage-gated activity in αON-sustained RGCs.

## Materials and methods

### Animals

All animal experiments described herein were approved by the Vanderbilt University Institutional Animal Care and Use Committee. We obtained adult (1.5–2 months old) male (*n* = 40) and female (*n* = 15) C57Bl/6 mice from Charles River Laboratories (Wilmington, MA, USA). Mice were housed at the Vanderbilt Division of Animal Care on a 12 h light/dark cycle and provided water and standard rodent chow as desired.

### IOP elevation and optic nerve crush

We measured IOP using TonoPen XL (Reichert Technologies, Depew, NY, USA), which was designed for use on human eyes. Previous studies indicate IOP measurements obtained from rodents using TonoPen correlate with direct cannulation measurements but requires a correction.^17,18^ We included corrected IOP measurements using the appropriate regression relationship between direct cannulation and Tonopen values from a previous study with cannulated IOP measurements.^18^ Additionally, our laboratory has shown reproducible IOP measurements from rodents with the TonoPen XL across many users, in line with the values we report here.^15–19,20,21,22,23,24,25^

TonoPen XL was calibrated each day prior to measuring IOPs according to the manufacturer’s specifications. The Ocu-Film cover (230651; Reichert Technologies) was used during measurements. Prior to performing IOP measurements, mice were anaesthetized (2.5% isoflurane). We recorded IOP of naïve eyes (baseline) on 2 separate days. We defined baseline IOP as the average of the two measurements of naïve eyes (Day 0). Following baseline IOP measurements, we unilaterally elevated IOP by injecting 1.5 μl of 15 μm polystyrene microbeads (Invitrogen, Carlsbad, CA, USA) into the anterior chamber; the fellow eye received an equal volume of sterile saline to serve as the control. We measured IOP two to three times per week for 1 week as described previously.^13–21,22,23–25,26,27^

We performed unilateral optic crush in anaesthetized [ketamine (112 mg/kg, KetaVed; Vedco Inc., St Louis, MO, USA)/xylazine (7 mg/kg, AnaSed injection; Akorn Inc., Lake Forest, IL, USA)] C57Bl/6 mice. After sedation, we provided analgesia through intraperitoneal injection of ketoprofen (5 mg/kg, Ketofen; Zoetis Inc., Parsipanny, NJ, USA). The optic nerve was exposed by performing a lateral canthotomy followed by a small incision in the conjunctiva. Afterwards, we used self-closing forceps (RS-5020; Roboz Surgical Instrument Co., Gaithersburg, MD, USA) to crush the optic nerve ∼1 mm posterior to the globe for 10 s. The contralateral nerve was also exposed but was not crushed. Afterwards, we applied triple antibiotic ointment (22373; Medique Products, Fort Myers, FL, USA) to the surgical site, and we monitored mice during their recovery from anaesthesia. Mice were kept on a heating pad both during surgery and while recovering to preserve body temperature.

### Anterograde axonal transport

We determined the fidelity of anterograde transport of RGC axons to their target neurons in the SC by measuring the transfer of cholera toxin subunit B (CTB) conjugated to Alexa Fluor 488 (CTB; Molecular Probes, Eugene, OR, USA). We intravitreally injected 1.5 μl of 1 μg/μl solution of CTB using a Hamilton syringe (7643-01; Hamilton, Reno, NV, USA) in anaesthetized mice (2.5% isoflurane). Two days after CTB injection, we transcardially perfused mice as described earlier.^15–19,20,1–5^ Following perfusion, we dissected out brains and cryoprotected in 20% sucrose solution. We then obtained coronal midbrain sections (50 µm) using a freezing sliding microtome (SM2000R; Leica Biosystems, Buffalo Grove, IL, USA). We imaged CTB fluorescence in alternating sections of the SC using a Nikon Ti Eclipse microscope. We quantified CTB signal intensity using a custom ImagePro (ImagePro 7; Media Cybernetics, Rockville, MD, USA) macro as described previously.^15^

### Optic nerve physiology

We measured mouse optic nerve compound action potentials using methods previously developed in our laboratory.^13–24^ We euthanized animals by cervical dislocation and decapitation. Afterwards, the top of the skull was removed, and we gently titled the brain upward to expose the optic nerves. We severed optic nerves at the optic chiasm and posterior to the optic nerve head. Once detached, we immediately placed optic nerves in ice-cold (4°C) carbogen-saturated (95% O_2_, 5% CO_2_) artificial cerebrospinal fluid (aCSF) for 30 min. The aCSF contained (in mM) 124 NaCl, 3 KCl, 2 CaCl_2_, 2 MgCl_2_, 1.25 NaH_2_PO_4_, 23 NaHCO_3_ and 10 glucose.^28^ The pH of the aCSF was 7.4. Optic nerves were equilibrated to physiological conditions for 30 min prior to recording. To prevent any order effects, we alternated the order we performed physiology on optic nerves.

We transferred optic nerves singly using a paint brush into a physiological recording chamber (Model PH1; Warner Instruments, Holliston, MA, USA) and continually perfused carbogen-saturated aCSF at a rate of 2 mL/min using a peristaltic pump (Model 7518; Masterflex, Vernon Hills, IL, USA). We maintained the aCSF solution at 35°C (Model TC-344C; Warner Instruments). The caudal end of the optic nerve was positioned into a recording suction electrode (Model 573040; A-M Systems, Sequim, WA, USA), and the rostral end of the optic nerve was positioned into a custom-made stimulating suction electrode. We fabricated electrodes with an average 350 μm diameter bore from borosilicate glass (Model TW150-4; WPI, Sarasota, FL, USA) using a puller system (Model P2000; Sutter Instruments, Novato, CA, USA). Recording and stimulating electrodes were fixed to separate micromanipulators (Model MM33; WPI). The stimulating electrode contained an Ag wire, and the recording pipette contained an Ag/AgCl^−^ wire; we filled both pipettes with aCSF. Optic nerves were stimulated (Model ISO-STIM 01-DPI; NPI Electronic, Germany) and evoked potentials were bandpass filtered (0.0001–10 kHz), amplified (100× gain, EXT-02-B; NPI Electronic), digitized (Digidata 1440A; Molecular Devices, San Jose, CA, USA) and sampled at 50 kHz (Clampex 10.6; Molecular Devices). Resistance between the nerve and recording pipette was measured by an electrode resistance meter (Model REL-08 B; NPI Electronic).

### RGC physiology

We performed whole-cell recordings from whole-mount mouse retinas as previously described.^19–21^ Animals were euthanized by cervical dislocation, eyes were enucleated, and retinas were dissected out under long-wavelength illumination (630 nm, 800 μW/cm^2^, FND/FG; Ushio, Cypress, CA, USA). Retinas were placed in carbogen-saturated Ames’ medium (US Biologic, Memphis, TN, USA) containing 20 mM glucose and 22.6 mM NaHCO_3_ (pH 7.4, 290 Osm). Retinas were mounted onto a physiological chamber, perfused with Ames’ medium at a rate of 2 ml/min and maintained at 35°C (Model TC-344C; Warner Instruments).

Retinal ganglion cells were visualized with an Andor CCD camera attached to an Olympus BX50 upright microscope using a 40× objective. For intracellular recordings, pipettes were constructed from borosilicate glass (Sutter Instruments) and filled with (in mM): 125 K-gluconate, 10 KCl, 10 HEPES (4-(2-hydroxyethyl)-1-piperazineethanesulfonic acid), 10 EGTA (ethyleneglycol-bis(β-aminoethyl)-N,N,N',N'-tetraacetic acid), 4 Mg-ATP, 1 Na-GTP (guanosine triphosphate) and 0.1 ALEXA 555 (Invitrogen). The pH of the intracellular solution was 7.35 and the osmolarity was 285 Osm. Resistances of pipettes containing intracellular solution were between 4 and 8 MΩ. Whole-cell signals were amplified (Multiclamp 700B; Molecular Devices) and digitized at a sampling rate of 50 kHz (Digidata 1550A; Molecular Devices). In a typical experiment, we measured resting membrane potential (RMP), light-evoked spike activity (full-field 365 nm, 3 s duration, pE-4000; CoolLED, Andover, UK) and voltage-gated responses to depolarizing current injections. Following completion of physiology experiments, retinas were fixed in 4% paraformaldehyde (PFA) for 24 h at 4°C.

### RGC immunohistochemistry and dendritic arbour analysis

After overnight fixation, retinas were immunolabelled for non-phosphorylated neurofilament H (SMI-32, 1:1000; BioLegend, San Diego, CA, USA) and choline acetyltransferase (ChAT, 1:500; Millipore, Burlington, MA, USA). Retinas were incubated in a blocking solution with 5% normal donkey serum for 2 h and then incubated for 3 days at 4°C with primary antibodies. We imaged RGCs *en montage* using an Olympus FV1000 inverted confocal microscope. RGC dendritic morphologies were hand traced in Adobe Photoshop, and we measured cross-sectional soma area, dendritic area, total dendritic length and number of branching points.^19,1–3^ The total dendritic length was defined as the sum of all dendritic lengths. A branch point was defined as the point of bifurcation of a dendrite from a parent dendrite. We also determined dendritic complexity using Sholl analysis (ImageJ version 1.53c), which measures the number of dendritic intersections with 10 μm concentric circles extending from the soma to distal dendritic tips (∼300 μm).

In a separate set of experiments, after IOP elevation or optic nerve crush, mice were transcardially perfused with phosphate-buffered saline (PBS) followed by 4% PFA. Whole retinas were dissected out and further post fixed in 4% PFA for 2 h at room temperature. We washed retinas 3× in PBS and incubated in blocking solution with 5% normal donkey serum. Retinas were incubated for 3 days at 4°C with the RGC selective marker RNA-binding protein with multiple splicing (1:200; PhosphoSolutions, Aurora, CO, USA) followed by incubation with secondary antibody for 2 h at room temperature. Whole retinas were imaged with a Nikon Ti Eclipse microscope (Nikon Instruments Inc., Melville, NY, USA) by a masked investigator. At least eight areas of each retina were used for cell counting. All cell counts were performed by a masked investigator.

### Optic nerve immunohistochemistry and node of Ranvier analysis

Mice were transcardially perfused with PBS and 4% PFA. Full-length optic nerves (from globe to optic chiasm) were then dissected. Optic nerves were post fixed in 4% PFA for 2 h, washed in PBS and then transferred to 30% sucrose for 3 days at 4°C. Optic nerves were placed in optimal cutting temperature medium and cryo-sectioned longitudinally. Nerve sections were washed 3× in PBS and then incubated in blocking solution containing 5% normal donkey serum for 2 h at room temperature. To visualize the crush site, optic nerve sections from CTB injected mice were incubated with glial fibrillary acidic protein (GFAP; 1:500; Abcam, Cambridge, UK) and counterstained with DAPI. Sections were incubated overnight at 4°C with primary antibodies Caspr1 (1:300; Millipore, Norwood, OH, USA), NaV1.6 (1:200; Millipore, Norwood, OH, USA) or NaV1.2 (1:300; Alomone Labs, Israel) followed by incubation with appropriate secondary antibodies for 2 h at room temperature. We verified NaV1.6 and NaV1.2 immunolabelling by preabsorbing tissue in blocking peptides prior to adding primary antibodies. Following immunohistochemical procedures, images were captured on an Olympus FV1000 confocal microscope using a 100× objective with a 2× zoom.

To analyse nodes of Ranvier, linear-segmented regions of interest (ROIs) were traced across the length of both paranodes in an individual axon segment as indicated by Caspr1 staining using ImageJ and ROIs were saved. The ROIs were then accessed by a Python script and ROI pixel values from corresponding NaV1.6 and Caspr1 channels were used to determine critical points (maxima, minima and region bounds). When plotted, Caspr1 shows a roughly bimodal distribution with maxima corresponding to the paranode region and minima corresponding to the node region. NaV1.6 staining shows a complementary pattern with a single maximum in the node region. A threshold was used to determine the boundaries of the node and paranode. Plots were generated for both the Caspr1 and NaV1.6 channels, critical points and the threshold line. These plots were manually checked for quality control, and low-quality plots were removed from the data prior to analysis. We measured the presence or absence of NaV1.2 labelling to determine the percent of NaV1.2-positive nodes quantified.

### Statistical analysis

We analysed and graphed data using Graphpad Prism (Version 9; Graphpad LLC, San Diego, CA, USA). Prior to quantification, we performed outlier (Grubb’s test) and normality tests (D’Agostino and Pearson test). For normally distributed data (alpha = 0.05), we performed parametric statistics (e.g. one-way ANOVA, *t*-test). If data sets failed normality tests, we performed non-parametric statistics (e.g. Kruskal–Wallis, Mann–Whitney). We identify the statistical test used for each analysis in the figure legends.

### Data availability

Data is available upon reasonable request from the corresponding author.

## Results

### Optic nerve crush increases axon excitability in the contralateral nerve

**Figure 1:**
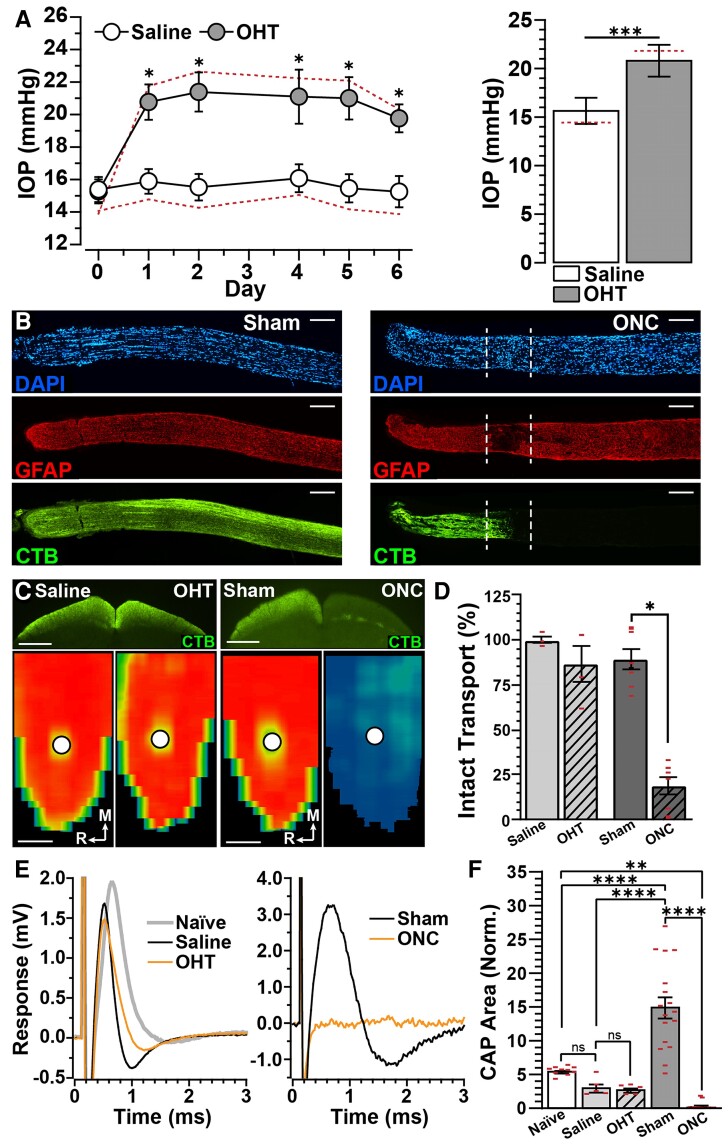
**Unilateral crush induces axonal hyperexcitability in the contralateral nerve.** (**A**, left) IOP elevation following a single unilateral injection of polystyrene microbeads (1.5 μl, *n* = 14) or saline (1.5 μl, *n* = 14; *P* < 0.05). (**A**, right) Mean IOP is elevated by 31% in microbead-injected eyes compared with saline controls (*P* < 0.001). Mean IOP corrected from a published regression based on cannulation measurements (red dotted line, see Reitsamer *et al*.^18^) resulted in a 5% increase in microbead IOPs and an 8% decrease in saline compared with our TonoPen XL measurements. (**B**) Longitudinal optic nerve sections immunolabelled for GFAP (middle panels) and counterstained with DAPI (blue) in (left) sham and (right) crush nerves. Intact CTB tracing indicates robust anterograde transport in sham nerves. In crushed nerves, deficits in CTB fluorescence indicates injury site (dashed lines). Scale bar = 200 µm. (**C**) CTB transport (top panels) to the SC and corresponding intensity heat maps (bottom) following (left) microbead injection and (right) ONC. Circles indicate optic discs. Scale bar = 500 µm. (**D**) Percentage of intact CTB transport to the SC is similar between eyes subjected to ocular hypertension (OHT) and saline injection (*P* = 0.55, *n* = 4). Transport is significantly diminished 1 week (1Wk) post ONC compared with sham controls (*P* < 0.0001, *n* = 8). (**E**) Representative current-evoked optic nerve compound action potential (CAP) traces from (left) naïve, saline, 1Wk OHT, (right) sham and 1Wk ONC . (**F**) Optic nerve CAP responses of naïve, saline and 1Wk OHT were statistically similar (*P* ≥ 0.67, *n* ≥ 5). ONC significantly reduced CAP responses compared with naïve (*P* = 0.007, *n* ≥ 10) and sham nerves (*P* < 0.0001, *n* = 17). Sham optic nerve CAP responses dramatically increased compared with naïve (*P* < 0.0001, *n* ≥ 10) and saline optic nerves (*P* < 0.0001, *n* ≥ 4). We normalized optic nerve CAP area to recording pipette resistance. Statistics: (**A**) Unpaired *t*-test. (**D** and **F**) One-way ANOVA and Tukey’s *post hoc* test. Significance indicators: *<0.05, **<0.01, ***<0.001, ****<0.0001. Data are presented as mean ± SEM.

Here, we compared pathology of RGCs and their axons using two models of optic neuropathy: the microbead occlusion model of glaucoma and optic nerve crush (i.e. traumatic optic neuropathy). For mice in the glaucoma cohort, we found TonoPen-measured IOP increased 1 day post-unilateral injection of microbeads and remained elevated for the duration of the experiment (+29.5%, *P* < 0.001, Fig. 1A, left). IOP of the affected eye significantly increased by 30.7% compared with saline-injected eyes (20.76 ± 1.09 versus 15.89 ± 0.76 mmHg, *P* = 0.005, Fig. 1A, right). When the mean values are corrected using a regression based on a published comparison with cannulation measurements for mice,^18^ the relative elevation in IOP increased to 51% (21.8 versus 14.4 mmHg). Despite these elevations in IOP, we did not detect RGC body dropout when compared with saline-injected eyes (*P* > 0.99, Supplementary Fig. 1). For animals in the traumatic optic neuropathy cohort, we confirmed injury site in longitudinal nerve sections by immunolabelling against GFAP, counterstaining nuclei with DAPI and tracking RGC active uptake and anterograde transport of CTB (Fig. 1B). Seven days post-unilateral optic nerve crush, we identified the injury site by the absence of GFAP labelling, aggregate DAPI-positive nuclei and loss of CTB transport by RGC axons. Degeneration caused by optic nerve crush was further evidenced by a significant loss of RGC bodies (*P* = 0.04, Supplementary Fig. 1). The contralateral eye, receiving sham surgery, appeared to maintain intact axons and RGC density (Fig. 1B, Supplementary Fig. 1).

We determined the influence of glaucoma or traumatic optic neuropathy on axon metabolic and electrical function by measuring RGC anterograde axonal transport of CTB to the SC and optic nerve compound action potential, respectively. After 1 week, axonal transport appeared intact in mice receiving saline and microbead injections, as well as in the sham surgery group (Fig. 1C). Optic nerve crush significantly reduced anterograde axonal transport of CTB to the SC by 78% compared with sham (*P* < 0.001, Fig. 1C and D).

Intact optic nerves produced a single peak in response to depolarizing current stimulation (Fig. 1E, left). We did not detect a significant difference between optic nerve compound action potentials of male and female mice (contralateral: *P* > 0.99, crush: *P* > 0.99). Therefore, we pooled these data. We found compound action potentials from nerves originating from saline-injected eyes similar to naïve nerves (3.14 ± 0.6 versus 5.57 ± 0.20 mV, *P* = 0.755, Fig. 1E, left, F).^24^ Following 1 week of IOP elevation, optic nerve compound action potential remained similar to that of saline-injected eyes (2.84 ± 0.31 versus 3.14 ± 0.6 mV, *P* > 0.99, Fig. 1E, left, F). However, this depolarizing response appeared to be eradicated following crush (Fig. 1E, right). Indeed, optic nerve crush significantly reduced compound action potentials (0.27 ± 0.13 mV) compared with potentials evoked from naïve (−95%, *P* = 0.006) and sham nerves (−98%, *P* < 0.001, Fig. 1E and F). Interestingly, we found the optic nerve compound action potential of the sham nerve increased by 170–377% compared with naïve and saline, respectively (*P* < 0.001, Fig. 1F).

**Figure 2:**
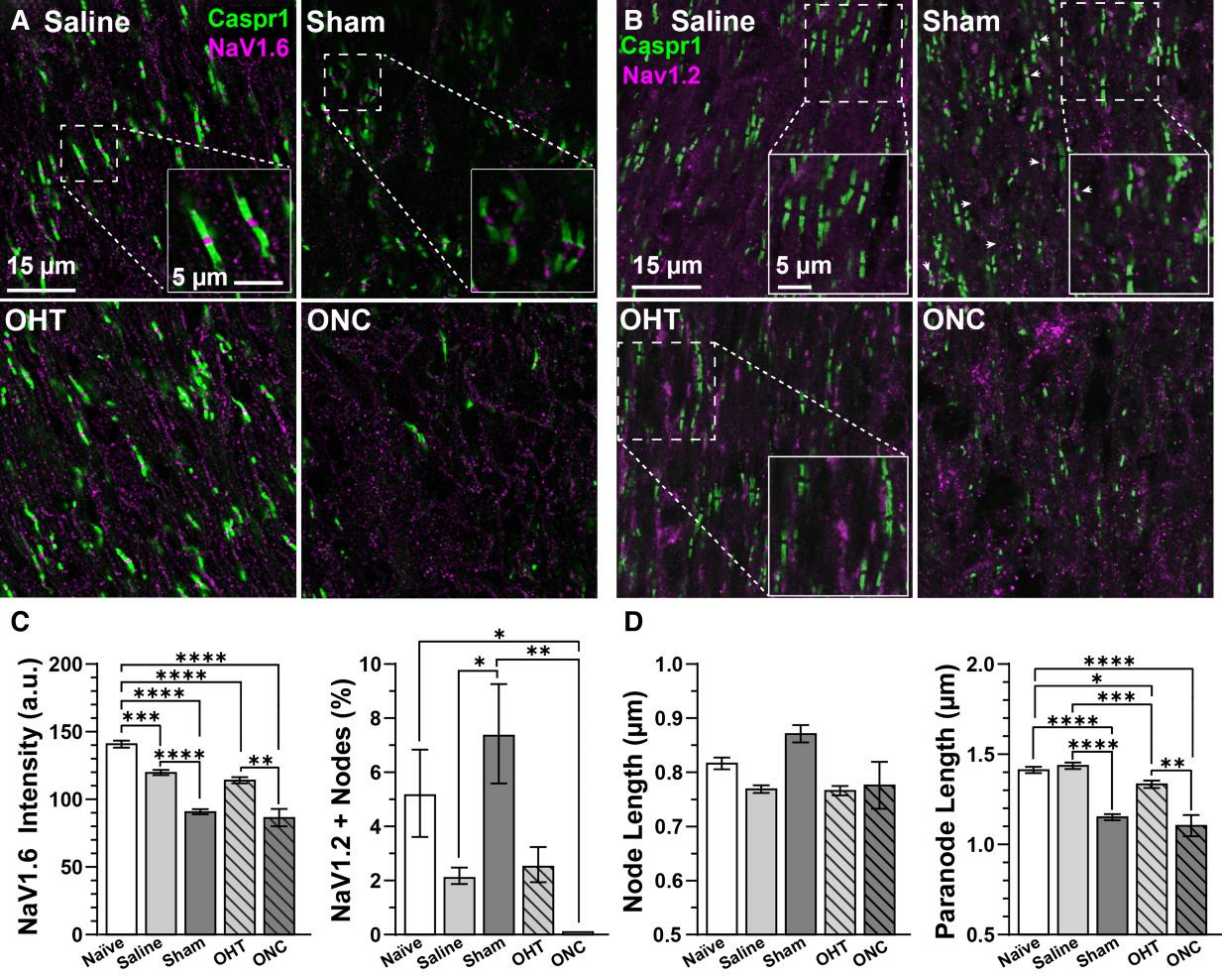
**Reduced expression of NaV1.6 and paranode morphology in experimental nerves.** Confocal micrographs of longitudinal optic nerve sections in mice subjected to saline- and microbead injection or sham surgery and ONC. (**A**) Immunostaining of Caspr1-labelled paranodes and Na_V_1.6 within nodes of Ranvier (scale bar = 15 µm). Insets are higher magnification micrographs showing example node-paranode complexes (scale bar = 5 µm). (**B**) Immunostaining of Caspr1-labelled paranodes and Na_V_1.2 (scale bar = 15 µm). Insets are higher magnification micrographs showing representative node-paranode complexes (scale bar = 5 µm). (**C, left**) Compared with nerves from naïve mice, NaV1.6 immunolabelling decreased in nerves from all experimental animals (*P* < 0.008, *n* ≥ 592 nodes, *n* ≥ 3 animals). After 1Wk ONC, Na_V_1.6 intensity also significantly diminished compared with optic nerves from 1Wk OHT mice (*P* = 0.0027, *n* ≥ 49, *n* ≥ 3 animals). (**C**, right) We did not detect a significant difference in NaV1.2 expression between naïve, saline, sham and OHT nerves. NaV1.2 expression significantly increased in sham nerves compared with nerves from saline-injected eyes (*P* = 0.0104). Crush significantly reduced NaV1.2 localization compared with naïve and sham nerves (*P* ≤ 0.0236). Naïve: *n* = 77 nodes, saline: *n* = 26 nodes, sham: *n* = 65 nodes, OHT: 29 nodes, ONC: 0 nodes. Animal *n* ≥ 3. (**D**, left) We did not detect a significant difference in node length between conditions (*P* ≥ 0.201). (**D**, right) Optic nerve axon paranode length of naïve and saline-injected eyes were similar (*P* > 0.99). Compared with naïve, paranode length significantly decreased in sham, OHT and ONC nerve (*P* ≤ 0.0205, *n* ≥ 49). OHT significantly reduced paranode extent relative to nerves from saline-injected eyes (*P* = 0.0006, *n* ≥ 509). Animal *n* ≥ 0.3 Statistics: (**C**, **D**) Kruskal–Wallis one-way ANOVA, Dunn’s *post hoc*. Significance indicators: *<0.05, **<0.01, ***<0.001, ****<0.0001. Data are presented as mean ± SEM.

Action potential regeneration is dependent on NaV channels accumulating within the nodes of Ranvier. Within mature nodes of Ranvier, NaV subunit 1.6 (NaV1.6) is the dominant isoform.^29^ Evidence suggest NaV1.6-mediated hyperexcitability is a subclinical indicator of neurodegenerative progression.^24,25–30^ Based on this premise, we determined if contralateral excitability is due to changes in nodal NaV1.6 expression flanked by paranodes identified by Caspr1 in longitudinal optic nerve sections (Fig. 2A).^24^ We found NaV1.6 accumulation significantly decreased in both sham and crushed nerves compared with nerves from naïve eyes (*P* ≤ 0.001, Fig. 2C, left). Furthermore, we observed a significant reduction in nodal NaV1.6 intensity in nerves from both saline- and microbead-injected eyes compared with nerves from naïve eyes (*P* ≤ 0.008; Fig. 2C, left). We verified NaV1.6 immunoreactivity by application of a blocking peptide prior to adding the primary antibody. We found NaV1.6 immunofluorescence reduced by the blocking peptide (Supplementary Fig. 2C and D). Based on these results, contralateral hyperexcitability does not appear to be due to increased NaV1.6 expression. Instead, NaV1.6 immunofluorescence decreased in injured and contralateral tissues compared with naïve.

Similar to our results, NaV1.6 immunolabelling is also reduced in optic nerve axons of experimental models of diseases that cause axon demyelination.^31,32^ In both acute and genetic models, demyelination induces the substitution of NaV1.6 for NaV subunit 1.2 (NaV1.2).^31,32^ Therefore, we determined if unilateral ocular hypertension or optic nerve crush altered the accumulation of NaV1.2 in optic nerve axon nodes of Ranvier. We did not detect a significant difference in NaV1.2 immunolabelling in optic nerve axons from naïve versus saline (*P* = 0.20) or naïve versus sham eyes (*P* = 0.58). However, the percent of NaV1.2-positive nodes in the contralateral nerve (8.1 ± 0.86%) significantly increased compared with nerves from saline-injected eyes (+273%, *P* < 0.0001, Fig. 2C, right). We saw NaV1.2 immunostaining within crushed optic nerves (Fig. 2B), but we did not detect NaV1.2 localized within nodes of Ranvier (0%, Fig. 2C, right). We confirmed NaV1.2 immunoreactivity by applying a blocking peptide before adding the primary antibody. We found NaV1.2 immunofluorescence diminished by the blocking peptide (Supplementary Fig. 2E and F). Based on these results, expression of NaV1.2 in nodes of Ranvier across naïve and injured tissues appears uncommon and variable, indicating ectopic expression of NaV1.2 is not a likely mechanism driving hyperexcitability in the sham nerve.

As mentioned above, decreased NaV1.6 expression in nodes of Ranvier indicates on-going demyelination.^32,33^ We further explored this possibility by measuring node and paranode length. Of course, we observed relatively few intact nodes of Ranvier in crushed nerves (Fig. 2A and B). We did not detect a significant difference in node length in nerves from naïve and experimental animals (*P* = 0.16; Fig. 2D, left). However, paranode length was reduced in both sham and crushed nerves compared with nerves from naïve and saline-injected eyes (*P* ≤ 0.002, Fig. 2D, right). Interestingly, we found 1 week of IOP elevation significantly reduced paranode length compared with nerves from naïve and saline-injected eyes (*P* ≤ 0.02, Fig. 2D, right). Our results suggest sham surgery, optic nerve crush and ocular hypertension induce demyelination near the nodes of Ranvier as evidenced by increased paranode length.

### Voltage-gated responses of αON-S RGC drive contralateral optic nerve hyperexcitability

**Figure 3:**
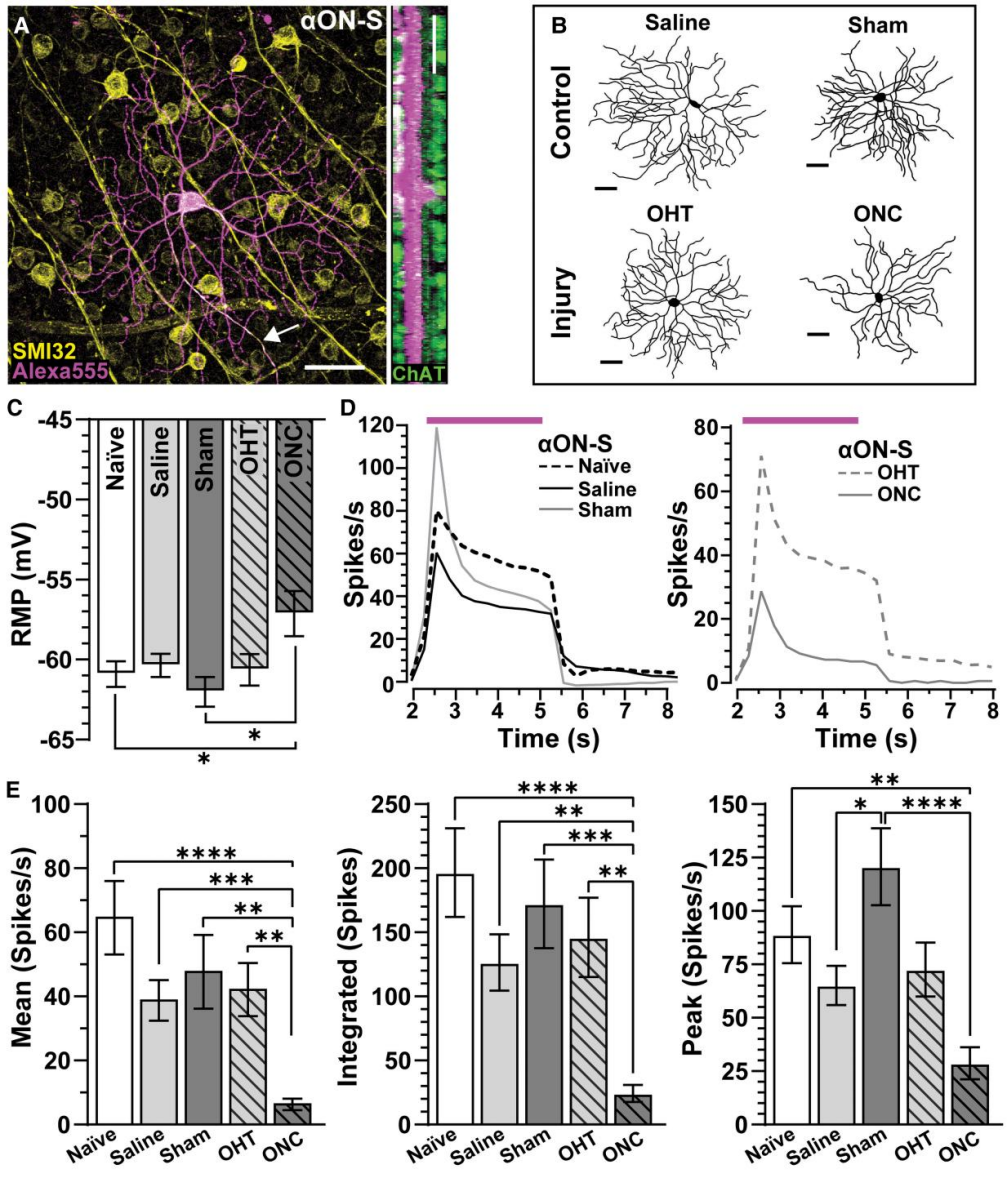
**Optic nerve crush depolarizes RMP and decreases light-evoked responses of αON-S RGCs.** (**A**) Confocal micrograph of Alexa 555 filled naïve αON-S RGC. Retinas were immunolabelled for SMI-32. Orthogonal rotation shows αON-S RGC dendrites ramify in the ON sublamina of the inner plexiform layer defined by choline acetyltransferase (ChAT). Arrow indicates RGC axon. Scale bars = 50 µm. (**B**) Reconstructed and skeletonized αON-S RGCs from saline, OHT, sham and ONC retinas. Scale bars = 50 µm. (**C**) ONC depolarized αON-S RGCs RMP compared with like cells from naïve (*P* = 0.05, *n* ≥ 16) and sham retinas (*P* = 0.03, *n* ≥ 11). (**D**, left) Averaged histograms (3 ms bins) of light-evoked response of naïve, saline and sham αON-S RGCs. (**D**, right) ONC reduced light responses of αON-S RGCs compared with OHT (right). (**E**) Compared with like cells from naïve retinas, light responses (mean, integrated and peak) of αON-S RGCs from sham and saline retinas were similar (*P* > 0.99, *n* ≥ 12). ONC significantly reduced light-induced responses (mean, integrated, peak) of αON-S RGCs compared with naïve (*P* < 0.0001, *n* ≥ 17). Peak light response is significantly greater in sham αON-S RGCs compared with saline αON-S RGCs (*P* = 0.02, *n* ≥ 12). After 1Wk, there is no difference in mean, integrated, or peak light responses for OHT αON-S RGCs compared with saline αON-S RGCs (*P* > 0.99, *n* ≥ 16). Statistics: (**C**) One-way ANOVA and Tukey’s *post hoc*. (**E**) Kruskal–Wallis one-way ANOVA, Dunn’s *post hoc*. Significance indicators: *<0.05, **<0.01, ***<0.001, ****<0.0001. Data presented as mean ± SEM.

Next, we investigated if contralateral optic nerve hyperexcitability originates from intraretinal mechanisms, and we directly compared pathology caused by IOP elevation versus optic nerve crush on RGCs by measuring physiologic responses to light and current stimulation. We identified two well-characterized alpha-type RGCs, αON- and αOFF-S RGCs, using physiological and morphological signatures. αON-S RGCs were determined based on heavy immunolabelling against SMI-32 and dendritic stratification within the inner plexiform layer marked by choline acetyltransferase. αON-S RGCs from naïve eyes possessed large somas (307.8 ± 15.2 µm2) with strong immunoreactivity to SMI-32 (SMI-32: background intensity = 6.9 ± 0.45), and their dendrites ramified within the proximal band of choline acetyltransferase-positive amacrine cell processes, defining the ON sublamina of the inner plexiform layer (Fig. 3A). After 1 week of IOP elevation, we found the morphology of αON-S RGCs similar to fellow cells from saline-injected eyes, but αON-S RGCs from sham and optic nerve crush eyes showed distinct signs of degeneration (Fig. 3B).

Physiologically, αON-S RGCs from naïve and saline eyes maintained similar RMPs (−60.9 ± 0.8 versus −60.4 ± 0.52 mV, *P* = 0.99; Fig. 3C), and these cells produced a sustained volley of action potentials during light onset (Fig. 3D, left). Similarly, RMP of αON-S RGCs from sham eyes (−62 ± 0.92 mV) was comparable with that of cells from naïve and saline eyes (*P* ≤ 0.79, Fig. 3C), but the light-evoked peak response of αON-S RGCs from sham eyes appeared to exceed that of cells from saline-injected eyes while the sustained component seemed similar (Fig. 3D, left). One week post crush, RMP of αON-S RGCs was significantly depolarized (−57.1 ± 1.4 mV) compared with αON-S RGCs in naïve and sham eyes (*P* < 0.05, Fig. 3C). Crush degraded both transient and sustained components of the light response of αON-S RGCs in the injured eye (Fig. 3D, right). When quantified, we found optic nerve crush significantly blunted the mean spike rate *P* ≤ 0.002), integrated spiking (*P* ≤ 0.0006) and light-evoked peak firing rate (*P* ≤ 0.004) of αON-S RGCs compared with like cells from naïve and sham eyes (Fig. 3E). Intriguingly, we found the light-evoked peak firing rate of αON-S RGCs from the sham eye significantly increased compared with cells from saline-injected eyes (120.7 ± 18 versus 65.2 ± 9.2 spikes/s, *P* < 0.01, Fig. 3E, right).

**Figure 4:**
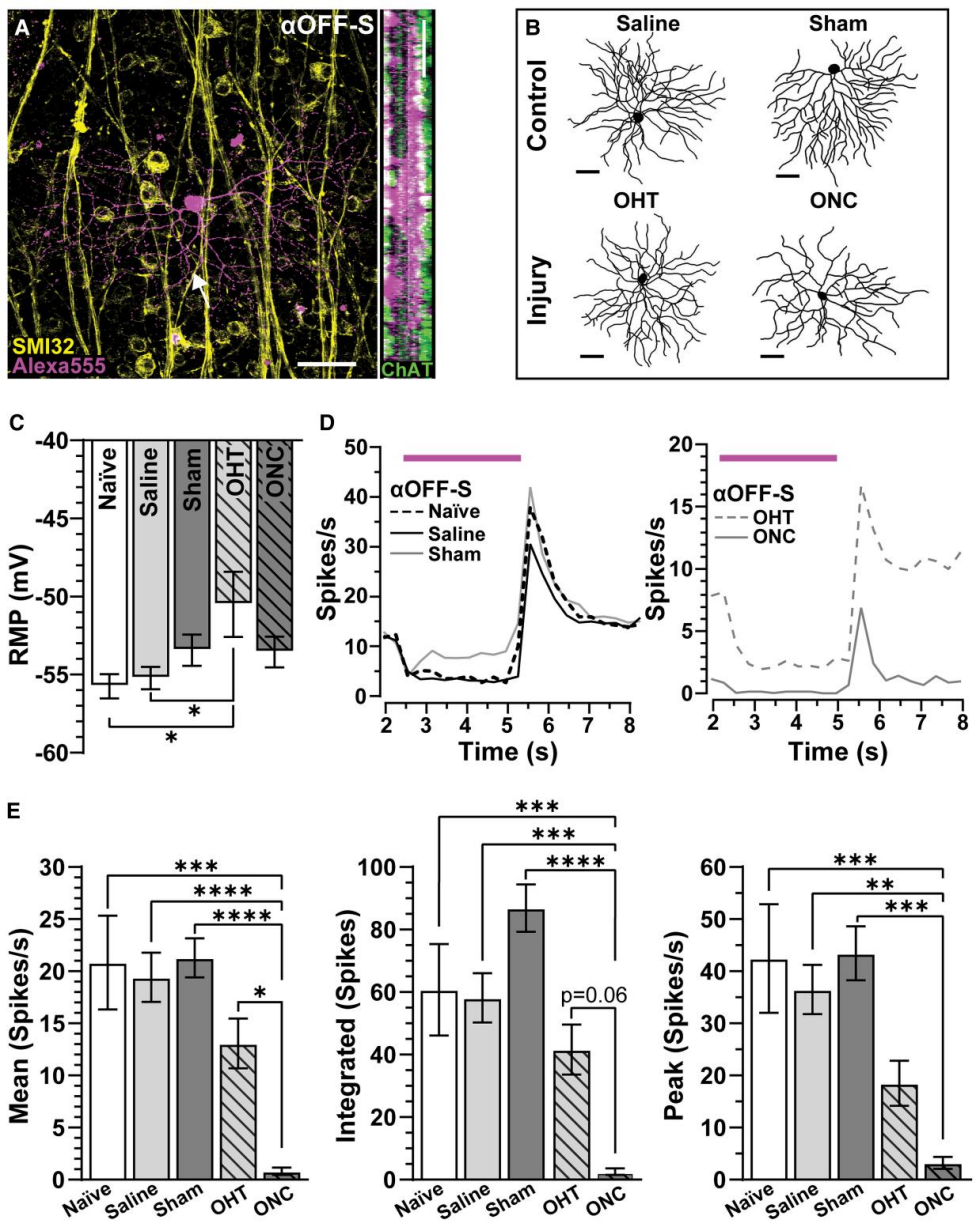
**Optic nerve crush diminishes light-evoked responses of αOFF-S RGCs.** (**A**) Confocal image of Alexa 555 filled αOFF-S RGC in a naïve retina immunolabelled against SMI-32. Orthogonal rotation shows αON-S RGC dendrites project in and beyond the OFF sublamina of the inner plexiform layer as defined by ChAT. Arrow shows the RGC axon. Scale bars = 50 µm. (**B**) Example αOFF-S RGCs, reconstructed and skeletonized, from saline, OHT, sham and ONC retinas. Scale bars = 50 µm. (**C**) OHT significantly depolarized αOFF-S RGCs RMP compared with like cells of naïve and saline retinas (*P* ≤ 0.023, *n* ≥ 8). (**D**, left) Averaged histogram of spontaneous and light-evoked spiking of αOFF-S RGCs from naïve, saline and sham retinas. (**D**, right) ONC reduced light responses of αOFF-S RGCs compared with OHT. (**E**) Light responses (mean, integrated and peak) of αOFF-S RGCs from naïve, saline and sham eyes were similar (*P* ≥ 0.98, *n* ≥ 12). ONC significantly blunted mean (*P* ≤ 0.0001), integrated (*P* ≤ 0.001) and peak (*P* ≤ 0.0003) light-driven responses for ONC αOFF-S RGCs compared with naïve and sham αOFF-S RGCs (*n* ≥ 10). Statistics: (**C**, **E**) One-way ANOVA and Tukey’s *post hoc*. Significance indicators: *<0.05, **<0.01, ***<0.001, ****<0.0001. Data are presented as mean ± SEM.

We defined αOFF-S RGCs by analysing dendritic stratification, SMI-32 immunolabelling and light responses. αOFF-S RGCs from naïve eyes possessed large somas (285.7 ± 19.97 µm2, Fig. 4A, left) with dendrites arbourizing just beyond the plexus of choline acetyltransferase-positive amacrine cell dendrites, marking the OFF sublamina of the inner plexiform layer (Fig. 4A, right). αOFF-S RGCs from naïve eyes produced immunoreactivity to SMI-32 (6.78 ± 0.96, Fig. 4A, left). The somato-dendritic compartment of αOFF-S RGCs from saline and sham eyes appeared similar, optic nerve crush appeared to decrease soma size and dendritic arbour complexity (Fig. 4B).

αOFF-S RGCs from naïve (−55.75 ± 0.78 mV), saline eyes (55.2 ± 0.71 mV) and sham (−53.44 ± 1.0 mV) maintained similar RMPs (*P* ≥ 0.48, Fig. 4C). In response to light onset, αOFF-S RGCs immediately hyperpolarized, suppressing the initiation of action potentials. Following light offset, these cells typically produced a robust pack of spikes (Fig. 4D). αOFF-S RGCs from naïve, saline and sham animals produced similar mean (*P* ≥ 0.98), integrated response (*P* ≥ 0.11) and peak light-induced firing rates (*P* ≥ 0.89, Fig. 4E). Crush significantly blunted αOFF-S RGC light-evoked spiking across all measurements relative to cells from naïve and sham animals (mean: *P* ≤ 0.0001; integrated: *P* ≤ 0.001; peak: *P* ≤ 0.007; Fig. 4E). Moreover, optic nerve crush modestly reduced the mean light-evoked spike rate of αOFF-S RGCs when compared with cells from microbead-injected eyes (*P* = 0.05, Fig. 4E).

**Figure 5:**
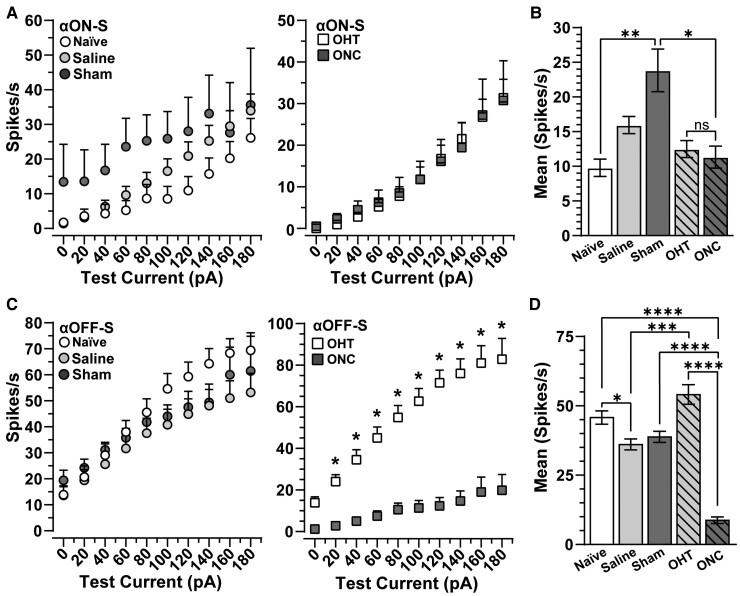
**Optic crush enhances current-evoked spiking in αON-S RGCs of the sham nerve.** (**A**, left) αON-S RGCs of sham nerves produce higher spike rates than like cells from naïve and saline eyes to depolarizing currents up to 140 pA (*n* ≥ 7). (**A**, right) Current-evoked spike rates of αON-S RGCs from ONC eyes are similar to αON-S RGCs from OHT eyes (right) (*n* ≥ 13). (**B**) αON-S RGCs from naïve and saline eyes produce similar mean current-evoked spike rates (*P* = 0.12). Mean current-evoked spike rate is significantly greater in sham αON-S RGCs compared with naïve αON-S RGCs (*P* = 0.002). (**C**, left) αOFF-S RGCs from naïve, saline and sham eyes produce similar spike rates in response to depolarizing currents up to 180 pA (*n* ≥ 12). (**C**, right) ONC reduced responses to depolarizing current in αOFF-S RGCs versus OHT αOFF-S RGCs (*P* ≤ 0.01, *n* ≥ 6). (**D**) Mean current-evoked spike rate is decreased for saline αOFF-S RGCs compared with naïve αOFF-S RGCs (*P* < 0.01). OHT increases αOFF-S RGC spike rate compared with saline αOFF-S RGCs (*P* < 0.0001). Conversely, ONC αOFF-S RGCs have a significantly lower mean current-evoked spike rate compared with sham αOFF-S RGCs (*P* < 0.0001). Statistics: (**A**, **C**) Two-way repeated measures ANOVA with Bonferroni’s *post hoc*. (**B**) Kruskal–Wallis one-way ANOVA, Dunn’s *post hoc*. (**D**) One-way ANOVA and Tukey’s *post hoc*. Significance indicators: *<0.05, **<0.01, ***<0.001, ****<0.0001. Data presented as mean ± SEM.

Next, we determine how intrinsic mechanisms of αON- and αOFF-S RGCs contribute to enhanced excitability of the optic nerve of sham eyes and degradation of compound action potentials of nerved subjected to crush by measuring spike output to depolarizing current injections in the absence of light. We found αON-S RGCs from naïve and saline eyes produced negligible spontaneous activity (0 pA test current: 1.6 ± 0.86 versus 1.43 ± 0.64 spikes/s, respectively) compared with cells from the sham eye (13.43 ± 10.85 spikes/s, *P* ≤ 0.05, Fig. 5A, left). Current-evoked responses of αON-S RGCs from sham eyes remained elevated versus naïve and saline, at smaller test currents (Fig. 5A, left). To the counter, responses to depolarizing current injections of αON-S RGCs from microbead-injected eyes and optic nerve crush eyes appeared strikingly similar (Fig. 5A, right). When averaged across current steps, we found spiking significantly increased in αON-S RGCs from sham eyes versus cells from naïve eyes by 143% (23.8 ± 3.1 versus 9.8 ± 1.2 spikes/s, *P* = 0.0015, Fig. 5B). However, unlike light-evoked responses (Fig. 3D and E), we did not detect a significant difference in average responses of αON-S RGCs from microbead-injected eyes and optic nerve crush eyes to current stimulation (*P* > 0.99, Fig. 5B). These results suggest mechanisms independent of those generating light responses of αON-S RGCs remain intact while pre- and post-synaptic mechanisms mediating light-evoked responses appear to falter 1 week after traumatic optic neuropathy. Moreover, enhanced excitability of αON-S RGCs from sham eyes mirrors hyperexcitability in the optic nerve compound action potential from sham animals.

Above, we observed injecting saline slightly increased average current-evoked spiking in αON-S cells, and sham surgery significantly enhanced excitability (Fig. 5A and B). For OFF-S RGCs, saline injection significantly depressed excitability compared with naïve cells (*P* = 0.006, Fig. 5C and D). Though also reduced, current-evoked responses of OFF-S RGCs from sham eyes were statistically comparable with cells from naïve eyes (*P* = 0.20, Fig. 5C and D). Interestingly, in corroboration with our previous findings,^3–5^ we found IOP elevation increased αOFF-S RGC excitability compared with like cells from saline-injected eyes (*P* < 0.001, Fig. 5C and D). On the other hand, responses of αOFF-S RGCs from saline-injected eyes decreased compared with naïve. Finally, optic nerve crush significantly reduced current-evoked responses of αOFF-S RGCs compared with naïve, sham and microbead-injected eyes (*P* ≤ 0.0001, Fig. 5C, right, D). In the context of our previous work,^19,3–5^ these results suggest intrinsic voltage-gated responses of αON-S RGCs drive contralateral optic nerve axon hyperexcitability following crush (Fig. 5A and B), and 1 week of ocular hypertension piques intrinsic mechanisms of αOFF-S RGCs, increasing excitability compared with like cells from saline-injected eyes (Fig. 5C and D). However, this increased excitability is relative to cells of the saline eye because either saline injection or stress induced by ocular hypertension reduced αOFF-S RGC excitability compared with naïve.

### Dendritic pruning is accelerated by optic nerve crush compared with ocular hypertension

**Figure 6:**
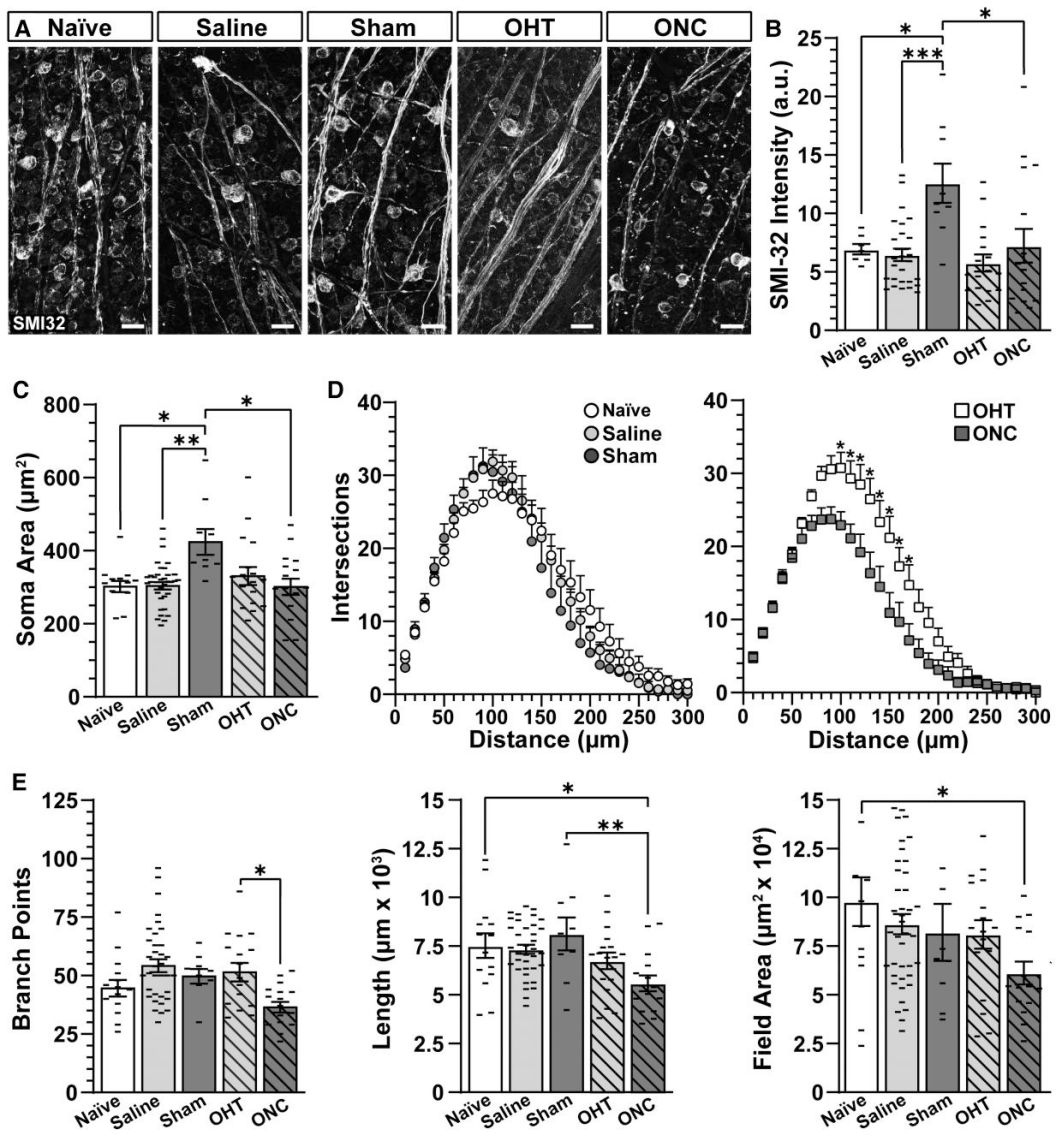
**Unilateral optic nerve crush produces bilateral pro-degenerative responses in αON-S RGCs.** (**A**) Representative confocal micrographs of SMI-32 immunolabelled whole-mount retinas. Scale bars = 25 µm. (**B**) Somatic SMI-32 intensity increased in sham αON-S RGCs compared with like cells from naïve (*P* = 0.04, *n* ≥ 7) and saline retinas (*P* < 0.001, *n* ≥ 9). ONC significantly reduced SMI-32 expression in αON-S RGCs compared with sham αON-S RGCs (*P* = 0.01, *n* ≥ 9). (**C**) Soma areas are larger in sham αON-S RGCs compared with naïve (*P* ≤ 0.02) and saline αON-S RGCs (*P* = 0.006, *n* ≥ 9). ONC decreased αON-S RGC soma area relative to sham (*P* = 0.02, *n* ≥ 9). (**D**, left) Sholl analysis indicates similarity in dendritic complexity of αON-S RGCs from naïve, saline and sham eyes (*P* = 0.16, *n* ≥ 9). (**D**, right) ONC significantly reduced dendritic intersections of αON-S RGCs compared with like cells from OHT eyes (*P* < 0.05). (**E**) αON-S RGCs from naïve, saline and sham retinas possess similar dendritic arbour morphologies based on branch points, dendritic length and field area (*P* ≥ 0.22, *n* ≥ 9). Total dendritic length is significantly reduced in αON-S RGCs following ONC compared with naïve (*P* < 0.05) and sham αON-S RGCs (*P* = 0.01, *n* ≥ 9). Dendritic field area is reduced by ONC in αON-S RGCs compared with naïve αON-S RGCs (*P* < 0.05, *n* ≥ 9). Statistics: (**B**, **C** and **E**) One-way ANOVA and Tukey’s *post hoc*. (**D**) Two-way repeated measures ANOVA with Bonferroni’s *post hoc* test. Significance indicators: *<0.05, **<0.01, ***<0.001, ****<0.0001. Data are presented as mean ± SEM.

We noted SMI-32 immunolabelling increased in αON-S RGCs from sham eyes, while SMI-32 immunostaining in αON-S RGCs from other conditions appeared similar to like cells from naïve eyes (Fig. 6A). As expected, neither saline injection, ocular hypertension, nor crush produced an appreciable change in αON-S RGC SMI-32 labelling compared with like cells from naïve eyes (*P* ≥ 0.96, Fig. 6B). However, at 1 week post crush, SMI-32 significantly accumulated in αON-S RGC somas from sham eyes (12.6 ± 1.67) compared with cells from naïve (6.9 ± 0.45) and saline eyes (6.5 ± 0.43, *P* ≤ 0.036, Fig. 6B). In line with this result, the cross-sectional area of αON-S RGC somas from sham eyes (429.5 ± 35.3 µm2) significantly increased 36–39% compared with αON-S RGCs from naïve (307.8 ± 15.2 µm2) and saline-injected eyes (309.7 ± 10.2 µm2, *P* ≤ 0.017) and fellow cells from eyes receiving optic nerve crush (307.1 ± 22.1 µm2, *P* = 0.019, Fig. 6C). Despite changes in somatic SMI-32 labelling and area, dendritic complexity of αON-S RGCs from sham eyes remained intact compared with cells from sham and saline-injected eyes as determined by the number of dendritic intersections (*P* ≥ 0.13, Fig. 6D, left), branch points (*P* > 0.91), total dendritic length (*P* = 0.76) and dendritic field area (*P* = 0.82, Fig. 6E). Optic nerve crush reduced the number of dendritic intersections (*P* ≤ 0.03, Fig. 6D, right) and branch points (−28%, 37.4 ± 2.2 versus 52.4 ± 3.9 branch points, *P* = 0.029) of αON-S RGCs in the directly affected eye compared with cells from microbead-injected eyes (Fig. 6E). As anticipated,^34^ these results suggest crush accelerates dendritic degeneration in αON-S RGCs compared with ocular hypertension. Additionally, our findings suggest unilateral lesion produces signatures of degeneration in αON-S RGCs of the contralateral, sham and eye.

**Figure 7:**
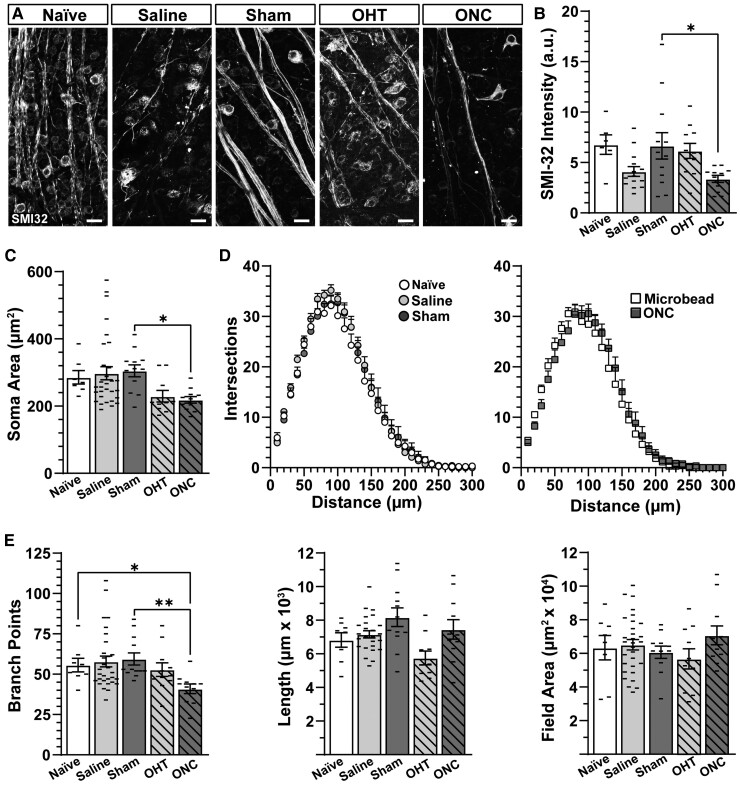
**Optic nerve crush piques mechanisms controlling dendritic branch points in αOFF-S RGCs.** (**A**) Example confocal images of SMI-32 immunolabelled whole-mount retinas. Scale bars = 25 µm. (**B**) ONC reduced SMI-32 accumulation in αOFF-S RGCs relative to sham αOFF-S RGCs (*P* < 0.05, *n* ≥ 11). (**C**) ONC decreased αOFF-S RGC soma area compared with like cells from sham eyes (*P* = 0.01, *n* ≥ 11). (**D**, left) Sholl analysis indicated the number of dendritic intersection is similar for αOFF-S RGCs from naïve, saline and sham eyes (*P* = 0.96, *n* ≥ 6). (**D**, right) The number of dendritic intersections is also similar for αOFF-S RGCs from ONC and OHT eyes (*P* = 0.48, *n* ≥ 10). (**E**) There is no difference in the number of branch points, dendritic length or dendritic field area of saline and sham αOFF-S RGCs compared with naïve (*P* ≥ 0.99, *n* ≥ 8). ONC significantly reduced the number of branch points in αOFF-S RGCs compared with like cells from naïve (*P* = 0.05, *n* ≥ 8) and sham retinas (*P* = 0.003, *n* ≥ 11). Statistics: (**B**) One-way ANOVA and Tukey’s *post hoc*. (**C** and **E**) Kruskal–Wallis one-way ANOVA, Dunn’s *post hoc*. (**D**) Two-way repeated measures ANOVA with Bonferroni’s *post hoc* test. Significance indicators: *<0.05, **<0.01, ***<0.001, ****<0.0001. Data are presented as mean ± SEM.

In contrast to αON-S RGCs, sham surgery did not appear to produce a significant change in SMI-32 expression in αOFF-S RGCs. However, optic nerve crush seemed to reduce SMI-32 expression in this cell type (Fig. 7A). Quantification of SMI-32 immunolabelling confirmed our observations: SMI-32 labelling in αOFF-S RGCs from sham eyes was comparable with that of αOFF-S RGCs from naïve and saline-injected eyes (*P* ≥ 0.147), and optic nerve crush significantly reduced SMI-32 labelling by 49% in αOFF-S RGCs versus like cells from sham eyes (3.37 ± 0.35 versus 6.65 ± 1.31, *P* = 0.047, Fig. 7B). Along with a reduction in SMI-32 expression, optic nerve crush significantly reduced soma area of αOFF-S RGCs by 28% compared with cells from sham eyes (218 ± 9.9 versus 305 ± 17.7 µm2, *P* = 0.01, Fig. 7C). Cross-sectional soma area of αOFF-S RGCs from saline and sham eyes was similar to αOFF-S RGCs from naïve eyes (*P* > 0.99).

Regarding dendritic arbour morphology, Sholl analysis indicated similar number of dendritic intersections in αOFF-S RGCs from naïve, saline and sham eyes (*P* ≥ 0.15, Fig. 7D, left). Interestingly, in contrast to αON-S RGCs (Fig. 6D, right), we found crush did not produce a significant change in the number of dendritic intersections when compared with like cells from microbead-injected eyes (*P* ≥ 0.93, Fig. 7D, right). However, compared with αOFF-S RGCs from naïve and sham eyes, optic nerve crushed significantly diminished the number of dendritic branching points (*P* ≤ 0.05, Fig. 7E). Similar to our results from Sholl analysis, neither optic nerve crush nor sham surgery significantly affected dendritic length (*P* > 0.99) or field area (*P* > 0.99) of αOFF-S RGCs compared with fellow cells from naïve eyes (Fig. 7E).

## Discussion

### Unilateral optic nerve trauma increased excitability of contralateral fibres through enhanced excitability of αON-S RGCs

Our main physiologic result is unilateral optic nerve trauma significantly enhanced excitability of fibres comprising the contralateral nerve in the absence of pathologic indications (Fig. 1B–F). In the context of previous findings from our laboratory and others, we suggest hyperexcitability is an early indicator of neurodegeneration, occurring prior to outright axon degeneration.^23,24,25–35,36^ This claim is further evidenced by neurodegenerative signatures, including reduced nodal NaV1.6 expression (Fig. 2A and C), reduced paranode length (Fig. 2D), enhanced accumulation of SMI-32 (Fig. 6A and B) and enlarged soma area of αON-S RGCs that send projections to the contralateral nerve (Fig. 6C).^31,32–37,38,39^ Based on these data, we propose unilateral trauma produces subtle indicators of degeneration in αON-S RGCs and their axons of the contralateral eye.

Enhanced excitability of the contralateral nerve is not due to alterations of nodal NaV1.6. In fact, NaV1.6 expression in contralateral nerve axons significantly decreased compared with nerves from naïve and saline-injected eyes (Fig. 2A and C). We found paranode length decreased in axons of the contralateral nerve versus nerves of naïve and saline-injected eyes (Fig. 2D). Collectively, these axonal stress responses indicate demyelination in the contralateral nerve, and all else being equal, would result in reduced compound action potentials.^31,32–39,40,41,42^

Our data indicates intraretinal mechanisms drive enhanced excitability in the contralateral nerve. αON-S RGCs from sham eyes produced greater firing rate to depolarizing current injections compared with responses of like cells from saline-injected and naïve eyes, respectively (Fig. 5B), exhibited increased accumulation of SMI-32 (Fig. 6A and B) and increased soma area (Fig. 6C). Enhanced excitability of αON-S RGCs within the sham eye appears to originate from intrinsic voltage-gated mechanisms. We found mean light-evoked responses and dendritic profiles of αON-S RGCs from sham eyes unchanged (Figs 3 and 6). However, in the absence of light, current-driven responses of these very cells increased. These findings suggest mechanisms of αON-S RGCs that contribute to spike initiation may be piqued by sham surgery or stress transduced from the contralateral, crushed and nerve.

While responses of αON-S RGCs from the sham eye increased, light- and current-evoked responses of αOFF-S RGC from the same eye remained intact (Figs 4D, E and 5C, D). Furthermore, αOFF-S RGCs projecting to the contralateral nerve exhibited similar SMI-32 accumulation, soma area and dendritic arbour profiles compared with like cells from naïve and saline-injected eyes (Fig. 7). Based on these data, the summed responses of αON- and αOFF-S RGC projections within the contralateral nerve would tilt towards hyperexcitability, which might be a compensatory response to loss of connections between RGC axons and central targets of the injured projection.^6^ This hyperexcitability in contralateral αON-SRGC may be due to increased NaV channels within unmyelinated axon segments, similar to our findings during early glaucoma.^25^

Several studies indicate immune cell activation in the contralateral nerve following unilateral injury.^7,8,9,10,11,12^ Hyperexcitability may be supported by reactive microglia that secrete pro-inflammatory cytokines such as tumour necrosis factor alpha (TNF-α).^43^ Interestingly, TNF-α increases NaV1.1, 1.2 and 1.6 channel mRNA expression and enhances NaV currents in cortical neurons.^44^ In the light of these data and our results, pro-inflammatory signals may prompt upregulation of NaV channels along unmyelinated axon segments of remaining RGCs that project through the contralateral nerve, inducing hyperexcitability.

Intriguingly, we also noticed 1 week of IOP elevation influences contralateral, saline-injected, tissues. We found immunolabelling for NaV1.6 within nodes of Ranvier significantly diminished, and current-evoked responses of αOFF-S RGCs reduced by ∼22% compared with naïve (Figs 2C and 5D). In contrast, IOP elevation tended to increase excitability of αON-S RGCs in saline-injected eye (+50%), although this result was not statistically significant relative to like cells from naïve eyes (Fig. 5B). Interestingly, both αON-S and αOFF-S RGCs have greatest density in the temporal-dorsal retina in mouse.^45^ Based on our physiologic results and topography of these RGC types, it might be the case that retrograde signals from central mechanisms differentially influence excitability in αON- and αOFF-S RGCs during unilateral injury as a form of compensation, and this compensation is exemplified in distinct RGCs with overlapping receptive fields that occupy similar topographical space.

### Pathologic progression of optic nerve trauma versus IOP elevation

Glaucomatous and traumatic optic neuropathy differ in both aetiology and rate of progression. However, evidence suggest common pathologic mechanisms that distinctly affect RGC bodies and axons.^19–46,47,48^ About 40 h post-optic nerve crush, axons distal to the injury-site fragment, while axons near the nerve head remain intact.^47^ A few hours later, axons proximal to the nerve head also fragment.^47^ At this time, the density of RGC bodies and axons is reduced by about 30%.^49^ Degeneration continues over time, and after 1 week, about 60% of RGC bodies and axons are lost (Fig. 1B and D, Supplementary Fig. 1).^49^

Previously, we have demonstrated progression of axonopathy and RGC degeneration during glaucoma, using the microbead occlusion model, by tracking deficits in anterograde axon transport of CTB to the SC, analysis of optic nerve axon density and RGC density. We found graduated deficits in RGC axon transport to the SC, optic nerve axon density and RGC bodies following 2–8 weeks of IOP elevation. Anterograde axon transport of CTB to the SC sharply declines by 40—50% after 2–4 weeks of IOP elevation, respectively.^25^ However, this duration of IOP elevation does not cause significant loss of RGC bodies or optic nerve axons.^19–21^ Following 5 weeks of ocular hypertension, anterograde axon transport of CTB remained reduced by half, but axon density significantly decreased by ∼20% while the retina maintained a full complement of RGC bodies.^20^ After 7–8 weeks of IOP elevation, transport of CTB to the SC is negligible and RGC body and axon density is reduced by about 50% (Fig. 1B and D).^15,49,50^ Collectively, these results indicate degeneration caused by optic nerve crush after 1 week is roughly equivalent to 2 months of IOP elevation.

One day after optic nerve crush, the density of nodes of Ranvier localized to axons distal and anterior to the injury site is reduced.^51^ After 7 days, nodes of Ranvier and constituent NaV1.6 are largely absent (Fig. 2).^51^ As a corollary, 5–7 days post crush, the optic nerve compound action potential is reduced by 65–95%, respectively (Fig. 1E and F).^52,53^ The visual evoked potential is reduced by ∼75% 1 week post crush.^54^

As expected, the influence of IOP elevation on axonal microarchitecture appears to be more subtle compared with crush. One week of IOP elevation modestly reduced paranode length and NaV1.6 immunolabelling (Fig. 2), and this level of reduction in nodal NaV1.6 localization appears to generalize 5 weeks post-microbead injection.^55^ However, following 1–2 weeks of IOP elevation by microbead occlusion, we found the optic nerve compound action potential remained intact (Fig. 1E and F).^24^ The influence of ocular hypertension by microbead occlusion on the optic nerve compound potential is unknown for later time points, but we have previously noted a modest reduction in the N1 component of the visual evoked potential after 4 weeks.^56^ After 2 months of IOP elevation, the visual evoked potential is significantly diminished by ∼45%.^57^ Overall, these results suggest the impact of optic nerve crush after 1 week on cortical vision is more pronounced compared with 2 months of IOP elevation.

Previous results indicate optic nerve crush reduces ganglion cell/nerve fibre layer thickness and the photopic negative response, which is largely generated by RGCs.^58,59^ Here, we refined this investigation by examining the influence of optic nerve crush and IOP elevation on the morphology and physiology of two well-characterized RGCs—the αON-S and αOFF-S RGCs.^19–60,61,62^ Following 1 week, optic nerve crush significantly depolarized RMP and impaired light-evoked voltage-gated responses of both αON-S and αOFF-S RGCs (Figs 3C–E, and 4C–E). Interestingly, we found depolarizing current-evoked responses of αON-S RGCs projecting to the crushed nerve comparable with responses of fellow cells from naïve eyes (Fig. 5Aand B). This finding contrasts with the influence of optic nerve crush on current-evoked responses of αOFF-S RGCs where optic nerve crush significantly reduced spiking in these cells (Fig. 5C and D). Our data support the notion that αON-S RGC, also known as the M4 intrinsically photosensitive RGCs, axons are less vulnerable to injury.^63,64,65,66^ Additionally, our results support the idea that OFF RGCs are more susceptible to injury compared with ON RGCs.^23–60,61–67,68,69^

One week of IOP elevation did not significantly affect light- or current-evoked responses of αON-S or αOFF-S RGCs when compared with like cells from naïve eyes (Figs 3–5), although RMP of αOFF-S RGCs was significantly more depolarized by ocular hypertension (Fig. 4C), a result similar to our previous findings.^3–5^ In earlier reports, we found 2 weeks of IOP elevation significantly depolarized RMP and increased light- and current-evoked responses of αON-S or αOFF-S RGC.^3–5^ This IOP-induced enhanced excitability appears to be due, in part, to increased accumulation of NaV1.6 channels along intraretinal ganglion cell unmyelinated axons.^25^ After 4 weeks of ocular hypertension, light-evoked excitability of both αON-S and αOFF-S RGCs decreased along with NaV1.6 and NaV1.2 immunolabelling within unmyelinated RGC axon segments.^2–5^ While the current-evoked excitability of αOFF-S RGCs decreased after 4 weeks of IOP elevation, responses of αON-S RGCs to depolarizing currents remained intact.^19^ Based on these results, mechanisms driving the light response of both ON and OFF RGCs appear to be most sensitive to optic nerve injury,^34,60,61^ and responses produced by unmyelinated axons of αON-S RGCs are less responsive to injury.

Morphologically, optic nerve crush significantly reduced αON-S and αOFF-S RGC dendritic branching (Figs 6D and 7D). In corroboration, others have also found RGC dendritic arbour complexity and post-synaptic density 95 labelling are significantly diminished 7 days post-optic nerve crush.^34^ Following 1 week of IOP elevation, we did not detect a significant change in dendritic arbour morphology in αON-S and αOFF-S RGCs (Figs 6 and 7). Two to 4 weeks of IOP elevation significantly reduced αON-S and αOFF-S RGC dendritic complexity.^19–21^ Therefore, dendritic arbour pruning appears to be an early response to optic nerve stress for both RGC types.

## Conclusion

These studies indicate unilateral optic neuropathy produces bilateral effects on intra- and extraretinal mechanisms controlling RGC dendritic excitation and axon excitability. Our results support the prevailing notion that solely using internal saline injection or sham surgery as controls is inadvisable. Moreover, our findings suggest susceptibility to injury is dependent on RGC type. Follow-up studies should determine the influence of anterograde versus retrograde signals on compensatory mechanisms evoked during degeneration.

## Supplementary Material

fcac251_Supplementary_Data

## Abbreviations

CTB =: cholera toxin subunit B
GFAP =: glial fibrillary acidic protein
IOP =: intraocular pressure
NaV =: voltage-gated sodium channel
ONC =: optic nerve crush
RMP =: resting membrane potential
RGC =: retinal ganglion cell
SC =: superior colliculus
SMI-32 =: non-phosphorylated neurofilament H.
