# Supplementary material for: Axon hyperexcitability in the contralateral projection following unilateral optic nerve crush in mice
Source: Brain Commun. 2022 Oct 3;4(5):fcac251. doi: 10.1093/braincomms/fcac251 (PMC9576152; doi:10.1093/braincomms/fcac251)
Supplement: fcac251_Supplementary_Data [file fcac251_supplementary_data.docx]

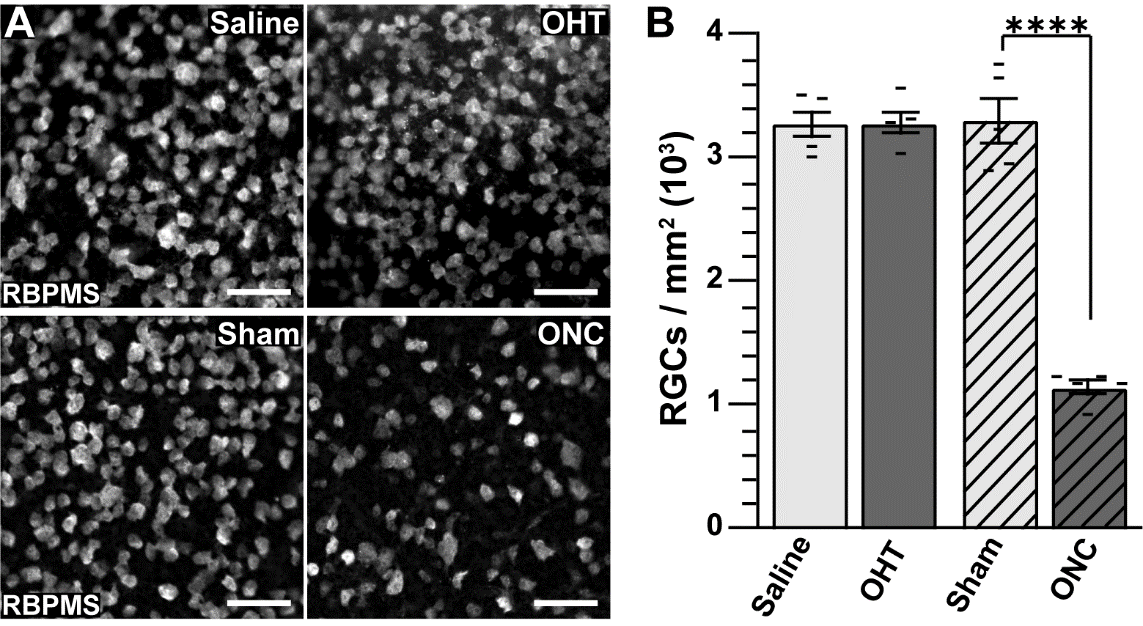


**Supplementary Figure 1. Optic nerve crush causes RGC drop out. (A)** Representative images of RBPMS immunolabeled whole-mount retinas. Scale bar = 50 µm. (**B**) There is no difference in the number of RBPMS+ RGCs in saline and sham retinas (p > 0.99). Similarly, following 1Wk OHT, there is no difference in RGC number compared to saline retinas (p > 0.99). However, with 1Wk ONC, there is a significant decline in the number of RBPMS+ RGCs (****p < 0.0001, n=5). Statistics: One-way ANOVA, Tukey post hoc comparison. Data presented as mean ± SEM.


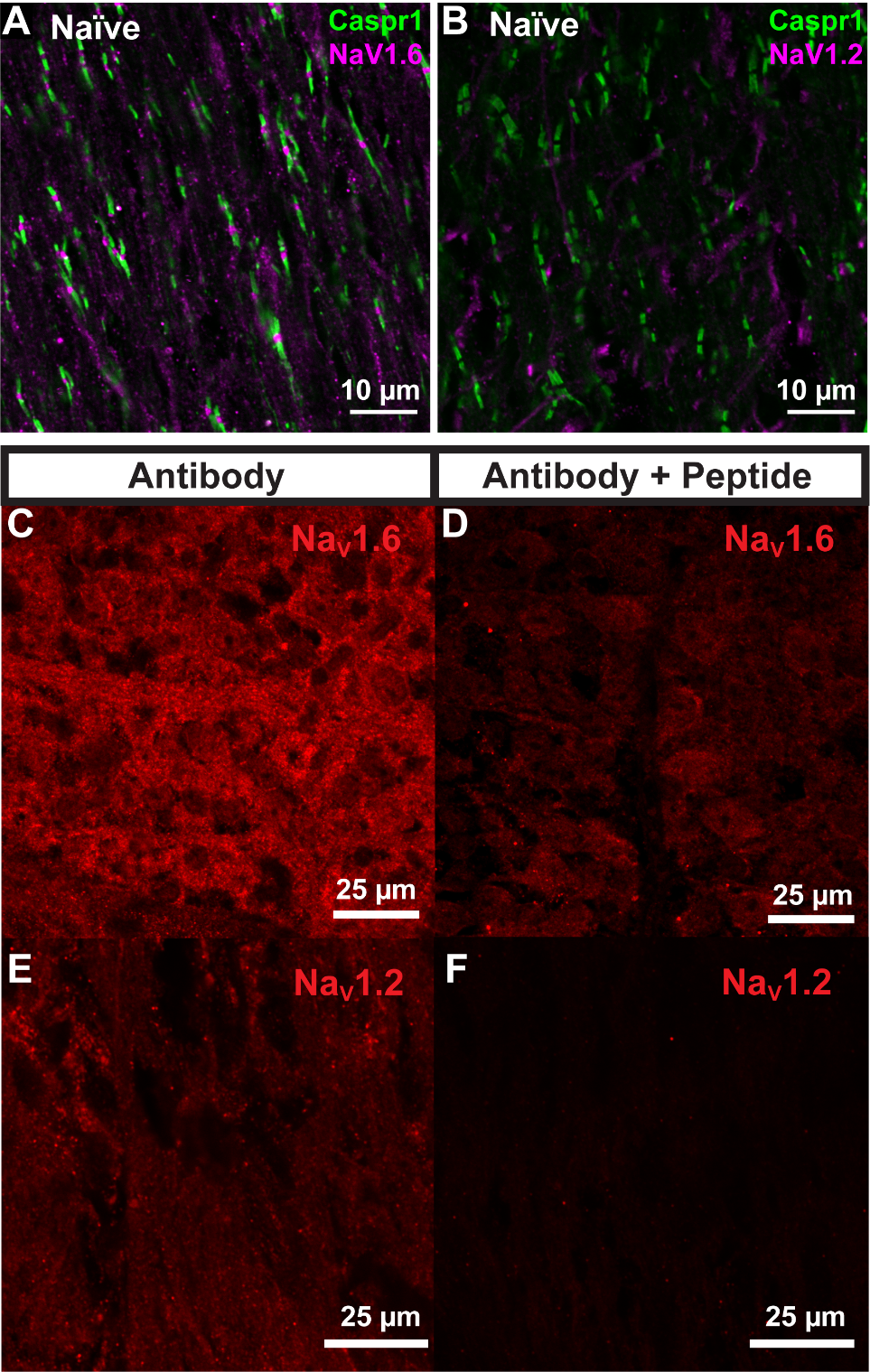


**Supplementary Figure 2. NaV immunolabeling in nerves from naïve mice and blocking peptide efficacy.** **(A)** NaV1.6 (magenta) and Caspr1 (green) immunolabeled node-paranode complexes in naïve optic nerves. **(B)** NaV1.2 (magenta) and Caspr1 (green) immunolabeled node complexes in naïve optic nerves. Scale bars = 10 µm **(C)** NaV1.6 immunoreactivity in whole-mount retinas, and **(D)** NaV1.6 immunolabeling following incubation in blocking peptide. **(E)** NaV1.2 immunoreactivity in longitudinal optic nerve slices, **(F)** and NaV1.2 immunolabeling after application of blocking peptide. Scale bars = 25 µm.
